# Post-Replication Repair Suppresses Duplication-Mediated Genome Instability

**DOI:** 10.1371/journal.pgen.1000933

**Published:** 2010-05-06

**Authors:** Christopher D. Putnam, Tikvah K. Hayes, Richard D. Kolodner

**Affiliations:** 1 Ludwig Institute for Cancer Research, University of California San Diego School of Medicine, La Jolla, California, United States of America; 2 Department of Medicine, University of California San Diego School of Medicine, La Jolla, California, United States of America; 3 Department of Cellular and Molecular Medicine, University of California San Diego School of Medicine, La Jolla, California, United States of America; 4 Cancer Center, University of California San Diego School of Medicine, La Jolla, California, United States of America; Brandeis University, United States of America

## Abstract

*RAD6* is known to suppress duplication-mediated gross chromosomal rearrangements (GCRs) but not single-copy sequence mediated GCRs. Here, we found that the *RAD6*- and *RAD18*-dependent post-replication repair (PRR) and the *RAD5*-, *MMS2*-, *UBC13*-dependent error-free PRR branch acted in concert with the replication stress checkpoint to suppress duplication-mediated GCRs formed by homologous recombination (HR). The Rad5 helicase activity, but not its RING finger, was required to prevent duplication-mediated GCRs, although the function of Rad5 remained dependent upon modification of PCNA at Lys164. The *SRS2*, *SGS1*, and *HCS1* encoded helicases appeared to interact with Rad5, and epistasis analysis suggested that Srs2 and Hcs1 act upstream of Rad5. In contrast, Sgs1 likely functions downstream of Rad5, potentially by resolving DNA structures formed by Rad5. Our analysis is consistent with models in which PRR prevents replication damage from becoming double strand breaks (DSBs) and/or regulates the activity of HR on DSBs.

## Author Summary

Genome instability is a hallmark of many cancers and underlies many inherited disorders that cause a predisposition to cancer. The human genome has many different types of duplicated sequences that can lead to genome instability by recombination-mediated pathways. We previously discovered that duplication-mediated chromosomal rearrangements are suppressed by a number of pathways. Some of these pathways were specific to rearrangements between genomic duplications. Here, we have performed a detailed analysis of pathways dependent upon *RAD6*, and have discovered that the error-free branch of post-replication repair (PRR) either is as an alternative to homologous recombination or prevents the generation of homologous recombination intermediates. Both of these functions could lead to genomic instability in the context of genomes containing substantial amounts of duplications. The extreme sensitivity of our assay to post-replication repair defects reveals substantial complexity in the interaction of PRR defects, suggesting the presence of many alternative PRR pathways. Together, the results emphasize the importance for appropriately balancing different repair pathways to maintain global genomic stability and highlight a number of defects that could underlie genome instabilities in some cancers.

## Introduction

Post-replication repair (PRR) was first identified in bacteria as a pathway for the repair of single-stranded gaps in DNA produced during the replication of DNA that had been damaged by exposure to ultraviolet light resulting in replication blocking lesions [1], [2]. PRR was also identified in the eukaryote *Saccharomyces cerevisiae* and found to be dependent on *RAD6* and *RAD18* [3]. PRR in both bacteria and eukaryotes is thought to not directly repair the replication-blocking lesions, but rather allows the replication machinery to bypass lesions. In eukaryotes, PRR has at least two downstream branches (reviewed in [4]). One branch extends nascent strands that are blocked by replication stalling lesions on the template strand using translesion or “error-prone” DNA polymerases, including DNA polymerase eta (Rev3-Rev7) and zeta (Rad30), which contribute to DNA damage-induced mutagenesis. The other “error-free” branch depends on *RAD5*, *MMS2*, and *UBC13* that is believed to allow extension by transiently pairing the blocked nascent strand and the other newly synthesized strand (“template-switching”). Template switching may occur by isomerization of the replication fork by Rad5 as demonstrated *in vitro* [5] and as first proposed thirty years ago [6], [7]. Alternatively, template switching might be mediated by a cross-fork template-switching mechanism proposed based on genetic similarities between *E. coli dnaK* mutants and *S. cerevisiae rad5* mutants and as suggested by the formation *RAD18*-, *RAD5-*, and *RAD51*-dependent double Holliday junctions in *sgs1Δ* mutants [8]–[10]. Importantly, these two models for template switching may not be mutually exclusive.

Many of the eukaryotic PRR genes encode proteins mediating protein ubiquitination [11], [12]. Rad6 is an E2 ubiquitin conjugase that is covalently linked by a thioester bond to the C-terminus of ubiquitin and transfers ubiquitin to targets recruited by the E3 ubiquitin ligases Bre1, Rad18 and Ubr1. Rad6 and Rad18 are required for PRR whereas Rad6-Bre1 mediates ubiquitination of histone H2B leading to transcriptional and checkpoint signaling [13]–[15] and Rad6-Ubr1 targets N-end rule substrates for degradation [16]. The Rad6-Rad18 complex monoubiquitinates PCNA at Lys164 [17] and the Rad17 subunit of the PCNA-like 9-1-1 checkpoint clamp at Lys197 [18]. Monoubiquitinated PCNA has been implicated in recruiting translesion polymerases [19], [20] as well as serving as substrate for synthesis of a Lys63-linked polyubiquitin chain by Mms2-Ubc13 E2 ubiquitin conjugase in conjunction with the Rad5 E3 ubiqutin ligase/DNA helicase [21], [22]. How the activities of Mms2 and Ubc13, and PCNA polyubiqutination channel DNA damage to error-free repair remains unclear [5]–[7].

In addition to roles in mediating tolerance to replication blocking DNA lesions, PRR genes have complex roles in maintaining genome stability. Both *rad5Δ* and *rad18Δ* mutants have elevated levels of spontaneous recombination [23] and rapid expansion of trinucleotide repeats [24]. Deletions of PRR genes appear to generally suppress gross chromosomal rearrangements (GCRs) mediated by single-copy sequences; *rad6Δ* suppresses the increased GCR rates caused by the *pif1-m2* allele [25] and deletion of *RAD5*, *RAD6*, *RAD18*, *UBC13* and *MMS2* similarly suppress the increased GCR rates caused by an *asf1* mutation [26]. In contrast, we found that deletion of *RAD6* dramatically increases the rate of GCRs mediated by homologous recombination (HR) between imperfect duplications resulting in increased accumulation of GCRs [27]. These differences likely reflect the fact that HR suppresses single copy sequence-mediated GCRs whereas HR produces duplication-mediated GCRs. Here we have sought to understand how the *RAD6* pathways function to specifically suppress duplication-mediated GCRs and to use the sensitivity of the duplication-mediated GCR assay to defects in *RAD6* to analyze interactions between components of *RAD6*-dependent pathways.

## Results

### *RAD6* suppression of duplication-mediated GCRs was epistatic to the replication stress checkpoint

**Figure 1:**
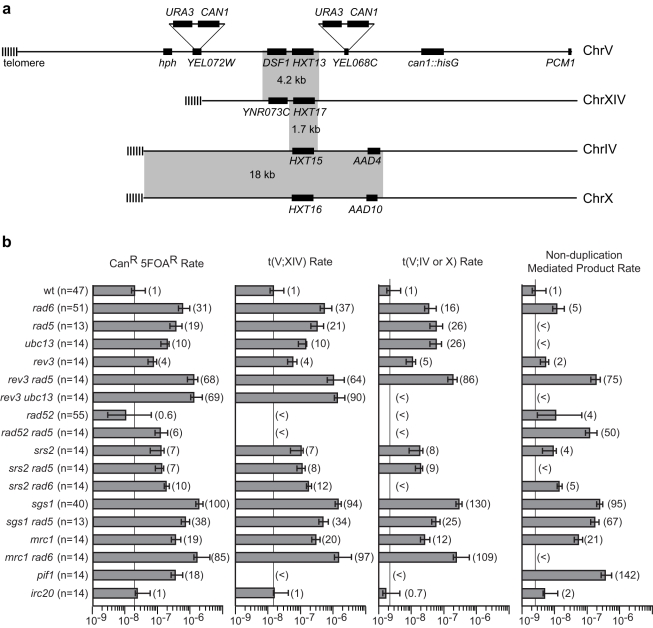
PRR defects result in increased rates of duplication-mediated translocations. **A.** The pre-duplication (*yel068c::CAN1/URA3*) and post-duplication (*yel072w::CAN1/URA3*) assays differ by whether or not they include the *DSF1-HXT13* homology in the breakpoint region (the left arm of chromosome V between the *CAN1/URA3* cassette and the most telomeric essential gene, *PCM1*). The hygromycin resistance marker is indicated by *hph*. Grey boxes indicate regions of homologies between the chromosomes. **B.** The rates of the total Can^R^ 5FOA^R^ product and the rates of t(V;XIV) and t(V;IV or X) translocations, and non-duplication-mediated GCR products in the *yel072w::CAN1/URA3* assay are depicted in a bar graph. Error bars indicate 95% confidence intervals and the fold increase for each rate is displayed in parentheses, (<) indicates that no isolates of that class were identified. The number of isolates analyzed is shown in parentheses after the genotype. The numerical GCR rates are presented in Tables 1, 2, 4 and 5.

Deletion of *RAD6* was previously found to specifically increase the spontaneous rate of duplication-mediated GCRs by comparing the rates of loss of a *CAN1/URA3* cassette on chromosome V in the *yel068c::CAN1/URA3* GCR assay, which lacks a duplication in the breakpoint region, with the *yel072w::CAN1/URA3* GCR assay, which contains the *DSF1-HXT13* duplicated region in the breakpoint region (Table 1; Figure 1a)[27]. The *DSF1-HXT13* region shares ∼4.2 kb of homology with chromosome XIV and ∼1.7 kb of homology with highly similar regions of chromosomes IV and X, and consequently most of the duplication-mediated GCRs are translocations between the *DSF1 HXT13* region on chromosome V and the homology regions on chromosomes XIV, IV and X. We analyzed the GCRs obtained in the *yel072w::CAN1/URA3* assay in the *rad6Δ* background and observed that the increased rates of forming homology-mediated t(V;XIV) and t(V;IV or X) translocations were responsible for most of the rate increases (Figure 1b). The majority of both homology and non-homology-mediated GCRs lost the telomeric end of chromosome V as determined by the loss of the telomeric hygromycin resistance marker (Table S1).

**Table 1:** Effects of combining *RAD6* and checkpoint gene mutations on duplication-mediated GCRs.

*Genotype*	*yel068c:: CAN1/URA3*		*yel072w:: CAN1/URA3*		*Ratio*
	*RDKY Number*	Can^R^5FOA^R^ Rate*	*RDKY Number*	Can^R^5FOA^R^ Rate*	
Wild-type**	6677	2.27 [1.3–4.8]×10^−9^ (1)	6678	1.97 [1.6–4.3]×10^−8^ (8.7)	8.7
*rad6Δ* **	6733	4.66 [0.0–17]×10^−9^ (2.1)	6750	6.03 [4.4–10]×10^−7^ (265)	130
*mrc1Δ* **	6730	3.35 [0.0–16]×10^−9^ (1.5)	6747	3.75 [2.8–5.2]×10^−7^ (165)	112
*rad6Δ mrc1Δ*	6901	1.76 [0.0–7.6]×10^−8^ (7.8)	6943	1.69 [1.3–4.4]×10^−6^ (744)	96
*tof1Δ* **	6767	5.71 [2.2–8.6]×10^−9^ (0.6)	6776	4.25 [2.3–5.9]×10^−7^ (187)	74
*rad6Δ tof1Δ*	6968	<2.25 [0.9–11]×10^−9^ (1.0)	6969	1.53 [1.1–2.0]×10^−6^ (674)	>678
*mrc1-aq* **	6766	1.51 [0.0–5.2]×10^−9^ (0.7)	6775	1.23 [0.6–5.3]×10^−7^ (54)	81
*rad6Δ mrc1-aq*	6966	6.07 [0.0–15]×10^−9^ (2.7)	6967	4.62 [3.0–6.8]×10^−7^ (203)	76
*mec1Δ sml1Δ* **	6760	2.34 [1.3–4.0]×10^−8^ (10)	6769	1.50 [0.5–2.7]×10^−7^ (66)	6.4
*rad6Δ mec1Δ sml1Δ*	6900	1.09 [0.7–1.9]×10^−7^ (48)	6942	2.12 [1.5–3.3]×10^−7^ (93)	1.9
*rad53Δ sml1Δ* **	6762	5.60 [2.5–11]×10^−8^ (25)	6771	3.05 [1.2–7.3]×10^−7^ (134)	5.4
*rad6Δ rad53Δ sml1Δ*	6904	1.06 [0.3–2.4]×10^−8^ (4.7)	6946	4.37 [2.0–7.9]×10^−7^ (193)	41
*rad9Δ* **	6765	2.17 [1.0–4.8]×10^−8^ (9.6)	6774	3.82 [0.0–10]×10^−8^ (17)	1.8
*Rad6Δ rad9Δ*	6903	1.87 [0.0–4.0]×10^−9^ (0.8)	6945	2.71 [2.1–3.4]×10^−7^ (119)	145

*The number in parentheses is the fold increase relative to RDKY6677. Numbers in brackets represent the 95% confidence intervals.

**Rates from [27].

Like *RAD6*, components of the replication stress checkpoint also has roles in specifically suppressing duplication-mediated GCRs [27]. To investigate the possibility that *RAD6* and the replication stress checkpoints function in the same pathway, we constructed double mutants containing a *rad6Δ* mutation along with different checkpoint defective mutations (Table 1). Remarkably, *rad6Δ* caused a synergistic increase in GCR rate that was statistically significant (p<0.0001 for the difference being due to chance) when combined with deletions of *MRC1* or *TOF1*, which encode signaling components of the replication fork that also have roles in sister chromatid cohesion [28], [29]. Comparison of the GCR products isolated from the *mrc1Δ* single mutant strain and *rad6Δ mrc1Δ* double mutant strain revealed that the increase in rate was primarily due to the formation of homology-mediated rearrangements (Figure 1b). Unlike *mrc1Δ*, however, *rad6Δ* appeared to be epistatic to the *mrc1-aq* allele (p = 0.1), which is specifically defective in the *MRC1* checkpoint function but not the replication function [30]. Similar to *mrc1-aq*, deletion of *MEC1* or *RAD53*, which encode protein kinases involved in the checkpoint response [31], appeared to be epistatic with *rad6Δ* (p = 0.09 and p = 0.4, respectively). In contrast, deletion of *RAD9*, which specifically impairs the DNA damage checkpoint but not the replication stress checkpoint [32], suppressed the rate of a *rad6Δ* mutation (p = 0.002), although the *rad6Δ rad9Δ* double mutant had a significantly higher duplication-specific GCR rate than the *rad9Δ* single mutant (p = 0.01). Taken together, these data suggest that *RAD6* functions in a pathway channeling replication damage away from duplication-mediated GCR formation in concert with replication stress checkpoint signaling and that deletion of *MRC1* and *TOF1* either causes increased replication errors that lead to GCRs or allows HR to target homology regions at dispersed chromosomal locations due to defects in sister chromatid cohesion that might restrict HR to sister chromatids.

### PRR is the major *RAD6*-dependent pathway that suppresses HR-dependent GCRs

To identify the *RAD6*-dependent pathways that suppress GCRs, each gene encoding a Rad6-associated E3 ubiquitin ligase was deleted in both the *yel068c::CAN1/URA3* and the *yel072w::CAN1/URA3* strain backgrounds. The *ubr1Δ* and *bre1Δ* mutations did not cause increased GCR rates in the *yel068c::CAN1/URA3* assay lacking a duplication (Table 2), consistent with previous results [25], [26]. Both mutations caused a small rate increase in the *yel072w::CAN1/URA3* duplication-mediated GCR assay, but these rates were substantially lower than that caused by deletion of *RAD6* (p<0.0001 for both). Deletion of *LGE1*, which encodes a protein that may function with Bre1 [33], was not distinguishable from deletion of *BRE1* in both GCR assays (p = 0.4). In contrast, the *rad18Δ* mutation, like a *rad6Δ* mutation caused little increase in the GCR rate in the *yel068c::CAN1/URA3* GCR assay but caused a large increase in the GCR rate in the *yel072w::CAN1/URA3* duplication-mediated GCR assay (Table 2). Thus, the Rad6-Rad18-dependent PRR branch appears to be the major pathway that functions in the *RAD6*-dependent suppression of duplication-mediated GCR formation.

**Table 2:** Effects of mutations in PRR subpathways on duplication-mediated GCRs.

*Genotype*	*yel068c:: CAN1/URA3*		*yel072w:: CAN1/URA3*		*Ratio*
	*RDKY Number*	Can^R^5FOA^R^ Rate*	*RDKY Number*	Can^R^5FOA^R^ Rate*	
Wild-type**	6677	2.27 [1.3–4.8]×10^−9^ (1)	6678	1.97 [1.6–4.3]×10^−8^ (8.7)	8.7
*rad6Δ* **	6733	4.66 [0.0–17]×10^−9^ (2.1)	6750	6.03 [4.4–10]×10^−7^ (265)	130
*bre1Δ*	6882	1.82 [0.0–6.3]×10^−9^ (0.8)	6924	4.89 [1.1–11]×10^−8^ (22)	26.8
*lge1Δ*	6889	<1.11 [0.9–1.6]×10^−9^ (<0.5)	6931	3.94 [2.3−5.0]×10^−8^ (17)	36
*ubr1Δ*	6923	<3.20 [2.8−6.1]×10^−10^ (<0.1)	6965	1.06 [0.8−1.6]×10^−7^ (47)	>331
*rad18Δ*	6905	2.14 [0.0−6.2]×10^−9^ (0.9)	6947	8.08 [0.0−12]×10^−7^ (356)	377
*taf14Δ (anc1Δ)*	6917	<1.37 [1.0−1.9]×10^−9^ (<0.6)	6959	2.02 [0.3−3.3]×10^−8^ (8.9)	15
*pol30-119*	6896	1.17 [0.3−2.5]×10^−8^ (5.2)	6938	6.39 [4.3−8.6]×10^−7^ (281)	55
*pol30-119 rad6Δ*	7033	<8.62 [0.0−31]×10^−10^(<0.4)	7036	3.71 [2.5−7.0]×10^−7^ (163)	>430
*srs2Δ* **	6741	7.18 [0.0−32]×10^−10^ (0.3)	6758	1.28 [0.6−1.6]×10^−7^ (56)	178
*srs2Δ rad6Δ*	7034	<1.35 [0.7−2.2]×10^−9^(<0.6)	7037	1.92 [1.7−2.4]×10^−7^ (163)	>142
*siz1Δ*	6915	3.13 [0.0−2.2]×10^−10^ (0.1)	6957	6.35 [2.4−9.1]×10^−8^ (28)	203
*rev3Δ*	6908	5.26 [0.0−25]×10^−10^ (0.2)	6950	7.59 [5.1−10]×10^−8^ (33)	144
*rad30Δ*	6907	1.15 [0.0−45]×10^−9^ (0.5)	6949	1.65 [0.9−2.1]×10^−7^ (73)	144
*rev3Δ rad30Δ*	6910	6.45 [4.8−9.7]×10^−10^ (0.3)	6952	9.26 [6.9−13]×10^−8^ (41)	144
*tsa1Δ*	6918	6.78 [3.1−11]×10^−9^ (3.0)	6960	1.30 [0.9−1.5]×10^−6^ (573)	192
*tsa1Δ rad30Δ*	6919	6.73 [2.0−12]×10^−9^ (3.0)	6961	6.39 [5.3−10]×10^−7^ (281)	95
*tsa1Δ rev3Δ*	6920	3.58 [1.1−5.4]×10^−8^ (16)	6962	1.65 [1.4−3.1]×10^−6^ (727)	46
*rad5Δ*	6898	5.00 [1.4−10]×10^−9^ (2.2)	6940	3.78 [2.4−5.8]×10^−7^ (167)	76
*mms2Δ*	6892	4.37 [1.6−18]×10^−9^ (1.9)	6934	2.47 [1.9−3.3]×10^−7^ (109)	57
*ubc13Δ*	6921	1.47 [0.0−3.1]×10^−9^ (0.6)	6963	2.06 [1.3−2.3]×10^−7^ (91)	140
*rev3Δ ubc13Δ*	6911	1.15 [0.6−3.8]×10^−8^ (5.1)	6953	1.35 [1.1−2.4]×10^−6^ (595)	117
*rev3Δ rad5Δ*	6909	4.36 [1.1−27]×10^−10^ (0.2)	6951	1.34 [0.9−1.8]×10^−6^ (590)	3062
*rad52Δ* **	6691	1.67 [1.0−2.7]×10^−8^ (7.4)	6708	1.09 [0.3−7.1]×10^−8^ (4.8)	0.7
*rad18Δ rad52Δ*	6906	9.95 [1.2−25]×10^−8^ (44)	6948	1.00 [0.5−1.7]×10^−7^ (44)	1.0
*rad5Δ rad52Δ*	6899	9.57 [6.4−19]×10^−8^ (42)	6941	1.26 [0.9−2.1]×10^−7^ (56)	1.3

*The number in parentheses is the fold increase relative to RDKY6677. Numbers in brackets represent the 95% confidence intervals.

**Rate from [27].

Monoubiquitination of PCNA by Rad6-Rad18 is an early event in PRR [17]. We therefore tested the *pol30-119* allele, which encodes a Lys164Arg mutant PCNA that lacks the PCNA ubiquitination site [17], and found that *pol30-119* caused essentially the same increase in the rate of duplication-mediated GCRs as caused by both *rad6Δ* and *rad18Δ* mutations (Table 2; p>0.01 and overlapping 95% confidence intervals for all pairwise comparisons). As the *pol30-119* allele also eliminates a major sumoylation site on PCNA [17], we also tested the effects of deleting *SIZ1*, which encodes a PCNA-modifying SUMO ligase, and *SRS2*, which encodes a helicase recruited to sumoylated PCNA [34], [35] that is also epistatic to PRR [36]. Neither of these deletions affected the rate of GCRs mediated by single copy sequences, consistent with previous data [25], [26]. Both *siz1Δ* and *srs2Δ* mutations caused a moderate increase in the rate of duplication-mediated GCRs, though the effect was significantly less than that caused by the *rad6Δ*, *rad18Δ* or *pol30-119* (p≤0.0001 for all pairwise comparisons). Consistent with this, the increased rates of duplication-mediated GCR products in the *srs2Δ* mutant were lower than that seen in the *rad6Δ* mutant (Figure 1b). Thus, the primary defect of the *pol30-119* allele in the suppression of duplication-mediated GCRs appears to be due to defects in PRR-mediated ubiquitination and rather than sumoylation. To confirm this, we analyzed *pol30*-*119 rad6Δ* double mutants and observed that the increased GCR rate seen in the *yel072w*::*CAN1*/*URA3* assay was indistinguishable from that caused by *pol30*-*119* and *rad6Δ* single mutations (Table 2; p>0.01 and overlapping 95% confidence intervals for all pairwise comparisons). We also analyzed the *srs2Δ rad6Δ* double mutants and observed a slight, but significant suppression of the *rad6Δ* duplication-mediated GCR rate (Table 2; p<0.0001), consistent with partial, but incomplete, epistasis of *RAD6* to *SRS2* for suppression of duplication-mediated GCRs.

### Defects in translesion polymerase-dependent PRR branch cause only moderate increases in HR-dependent GCRs

To understand which PRR branch suppresses duplication-mediated GCRs, we first analyzed the role of the translesion polymerases. Deletion of *REV3*, encoding the catalytic subunit of DNA polymerase zeta, and *RAD30*, encoding DNA polymerase eta, caused very small increases in the rate of single copy sequence-mediated GCRs (Table 2), consistent with previous results [25], [26], [37], but both caused moderate increases in the rate of duplication-mediated GCRs, although the rates were not increased to the extent seen for *rad6Δ* or *rad18Δ* mutations (Table 2). Moreover, the *rev3Δ* mutation caused roughly equivalent fold increases in the rates of forming both duplication-mediated and non-duplication-mediated GCR products (Figure 1b). The *rev3Δ rad30Δ* double mutant also had a moderate increase in the rate of duplication-mediated GCRs that was indistinguishable by 95% confidence intervals to the rate caused by the *rev3Δ* and *rad30Δ* single mutants, suggesting involvement in a single genetic pathway consistent with biochemical experiments demonstrating that DNA polymerases eta and zeta function sequentially to bypass specific lesions [38]. Deletion of *TSA1*, which encodes a thioredoxin peroxidase that suppresses oxidative damage of DNA in *S. cerevisiae* [37], [39] caused over a 65-fold increase in the rate of duplication-mediated GCRs (Table 2). Surprisingly, the *rev3Δ* and *rad30Δ* mutations did not cause synergistic interactions in either GCR assay when combined with a *tsa1Δ* mutation. This lack of a synergistic interaction is consistent with previous results obtained for GCR rates in single-copy sequences [37]. This suggests that the translesion polymerases either do not repair or bypass the oxidative damage that leads to duplication-mediated GCRs in *tsa1Δ* mutants or that other repair pathways, such as base-excision repair, nucleotide-excision repair or mismatch repair [39], [40], can efficiently repair *tsa1Δ*-mediated damage in the absence of *REV3* or *RAD30*.

### Defects in the error-free PRR branch caused large increases in HR-dependent GCRs

Deletion of *RAD5*, *MMS2* or *UBC13* that function in the error-free PRR branch as well as deletion of *RAD6* and *RAD18* did not cause a substantial increase in the rate of single copy sequence-mediated GCRs (Table 2). These results were consistent with those of one previous study [26], but the results for *rad5Δ* and *rad18Δ* were inconsistent with the results of another study that reported that *rad5Δ* and *rad18Δ* mutations caused an increase in the rate of single copy sequence-mediated GCRs [25]. Despite this, all of these deletions caused a significant increase in the rate of duplication-mediated GCRs (Table 2), which suggests that the error-free branch, and not the translesion polymerase branch, is the major PRR pathway that suppresses duplication-mediated GCRs. The rate increases caused by *rad5Δ* and *ubc13Δ* mutations in the *yel072w::CAN1/URA3* assay were due to increased rates of formation of the t(V;XIV) and t(V;IV or X) non-reciprocal translocations (Figure 1b). Deletion of *TAF14 (ANC1)*, which has been reported to be epistatic to *RAD5* in the repair of alkylation damage [41] and which encodes a protein involved in the RNA polymerase II-associated complexes TFIID, TFIIF, RSC, SWI/SNF, INO80, NuA3, and Mediator [42], had no effect on the GCR rate in either the single copy sequence- or duplication-mediated GCR assays (Table 2). Double mutants including the *rev3Δ ubc13Δ* and *rev3Δ rad5Δ* double mutants in which both the translesion polymerase and error-free PRR branches were defective had low GCR rates in the *yel068c::CAN1/URA3* assay. In contrast, these double mutants had increased rates of duplication-mediated GCRs that were significantly higher than seen in either single mutant individually but were not significantly different (by their 95% confidence intervals) from the rates of duplication-mediated GCRs seen in the *rad6Δ* and *rad18Δ* single mutants (Table 2). The t(V;XIV) duplication-mediated GCR product dominated the GCR products obtained in the *yel072w::CAN1/URA3* assay in the *rev3Δ ubc13Δ* and *rev3Δ rad5Δ* mutants; however, increases in the rates of t(V;IV or X) translocations and non-duplication-mediated GCRs were also observed in the *rev3Δ rad5Δ* double mutant (Figure 1b).

A *rad52Δ* mutation eliminated the increased rate of duplication-mediated GCRs rate due to the *DSF1-HXT13* duplication in the *yel072w::CAN1/URA3* assay caused by the *rad18Δ* and *rad5Δ* mutations (Table 2), consistent with the previously determined role of *RAD52* in the formation of duplication-mediated GCRs [27]. Similarly, no homology-mediated translocations were observed among the GCRs identified in the *yel072w::CAN1/URA3* GCR assay in the *rad52Δ* single or *rad5Δ rad52Δ* double mutants (Figure 1b). Remarkably, the *rad18Δ rad52Δ* and *rad5Δ rad52Δ* double mutants showed a synergistic increase in the rate of single copy sequence-mediated GCRs in the *yel068c::CAN1/URA3* assay (Table 2; p = 0.005 and p<0.0001, respectively), suggesting that PRR and HR are redundant in suppressing single-copy sequence-mediated GCRs such as *de novo* telomere additions and chromosome fusions that occur in the absence of extensive homology targets [43], [44]; consistent with this, the rate of non-duplication-mediated GCRs in the *yel072w::CAN1/URA3* assay was increased 50-fold in the *rad5Δ rad52Δ* double mutant compared to 5-fold and <11-fold increases in the *rad52Δ* and *rad5Δ* single mutants, respectively (Figure 1b).

### The Rad5 helicase activity, but not its RING-finger domain, suppresses duplication-mediated GCRs

Rad5 is a DNA helicase as well as an E3 ubiquitin ligase; both activities are required for the function of Rad5 in repair of UV damage by PRR [45]. Thus, we tested the ability of *RAD5* plasmids containing different *rad5* mutations to complement the defects in suppressing duplication-mediated GCRs caused by a *rad5Δ* mutation (Table 3). In contrast to the effects of *rad5* mutations on UV damage, we found that a *rad5* mutant plasmid containing RING finger mutations (C914A C917A) was able to significantly complement the *rad5Δ* mutant (p<0.0001). However, the GCR rate seen with the RING finger plasmid was 2-fold higher than that seen with a plasmid bearing a wild-type *RAD5* gene, which is a small but significant increase (p = 0.009). In contrast, a *rad5* mutant plasmid with defects in the Walker B motif of the helicase domain (D681A E682A) resulted in an increase in the rate of duplication-mediated GCRs similar to that of the empty vector control (p = 0.4). These results indicate that the helicase activity of Rad5, in contrast to its RING finger-dependent E3 ubiquitin ligase activity, is the most important Rad5 activity required for the *RAD5*-dependent suppression of duplication-mediated rearrangements.

**Table 3:** Complementation of *rad5Δ* in the *yel072w::CAN1/URA3* assay.

*Plasmid allele*	*Can^R^ 5FOA^R^ Rate*
*RAD5*	7.48×10^−8^ (1)
*none*	1.08×10^−6^ (14.4)
*rad5-C914A C917A*	1.50×10^−7^ (2.0)
*rad5-D681A E682A*	1.01×10^−6^ (13.5)

### *RAD5* has *RAD6*- and *UBC13*-independent but PCNA Lys164-dependent roles in suppressing duplication-mediated GCRs

*RAD5* has complex genetic relationships with other PRR genes. *RAD5* has *MMS2*- and *UBC13*-independent roles in the repair of UV damage that is processed through the action of translesion polymerases [45], [46]. *rad5Δ* single mutants also have higher UV sensitivity than *ubc13Δ* and *mms2Δ* single mutants [45], and the *rad5Δ mms2Δ* double mutant is more sensitive to DNA damaging agents than either single mutant [47]. We therefore tested the *rad5Δ ubc13Δ* double mutant and found that like the single mutants, the double mutant did not have an increased rate of single copy sequence-mediated GCRs in the *yel068c::CAN1/URA3* assay (Table 4). In contrast, the double mutant had an increased rate of accumulating duplication-mediated GCRs in the *yel072w::CAN1/URA3* assay compared to the respective single mutants (p = 0.0004 relative to *rad5Δ* and p<0.0001 relative to *ubc13Δ*), although this increased GCR rate could not be distinguished from that of *rad6Δ* and *rad18Δ* single mutants or *rev3Δ rad5Δ* and *rev3Δ ubc13Δ* double mutants (Table 2; p = 0.2 and p = 0.4 respectively). These results suggest that *RAD5* and *UBC13* may have some independent roles in suppressing duplication-mediated GCRs.

**Table 4:** Effects of combining *rad5Δ* with mutations in *RAD6*-pathway genes on duplication-mediated GCRs.

*Genotype*	yel068c:: CAN1/URA3*		yel072w:: CAN1/URA3*		*Ratio*
	*RDKY Number*	Can^R^5FOA^R^ Rate*	*RDKY Number*	Can^R^5FOA^R^ Rate*	
Wild-type**	6677	2.27 [1.3−4.8]×10^−9^ (1)	6678	1.97 [1.6−4.3]×10^−8^ (8.7)	8.7
*rad5Δ*	6898	5.00 [1.4−10]×10^−9^ (2.2)	6940	3.78 [2.4−5.8]×10^−7^ (167)	76
*ubc13Δ*	6921	1.47 [0.0−3.1]×10^−9^ (0.6)	6963	2.06 [1.3−2.3]×10^−7^ (91)	140
*ubc13Δ rad5Δ*	6922	5.22 [4.8−36]×10^−10^ (0.2)	6964	9.42 [6.4−13]×10^−7^ (415)	1803
*rad6Δ*	6733	4.66 [0.0−17]×10^−9^ (2.1)	6750	6.03 [4.4−10]×10^−7^ (265)	130
*rad6Δ rad5Δ*	6902	1.57 [0.5−8.1]×10^−9^ (0.7)	6944	1.65 [1.1−3.7]×10^−6^ (727)	1052
*pol30-119*	6896	1.17 [0.3−2.5]×10^−8^ (5.2)	6938	6.39 [4.3−8.6]×10^−7^ (281)	55
*Pol30-119 rad5Δ*	6897	8.58 [4.2−19]×10^−9^ (3.8)	6939	4.93 [3.9−6.2]×10^−7^ (217)	57
*siz1Δ*	6915	3.13 [0.0−2.2]×10^−10^ (0.1)	6957	6.35 [2.4−9.1]×10^−8^ (28)	203
*Siz1Δ rad5Δ*	7035	6.15 [0.0−52]×10^−10^ (0.3)	7038	5.75 [4.5−8.8]×10^−7^ (253)	935

*The number in parentheses is the fold increase relative to RDKY6677. Numbers in brackets represent the 95% confidence intervals.

**Rate from [27].

To investigate the possibility that *RAD5* has some functions in suppressing duplication-mediated genome rearrangements that are independent of PRR, we tested the *rad5Δ rad6Δ* and *rad5Δ pol30-119* double mutants. The *rad5Δ rad6Δ* double mutant had an increased rate of accumulating duplication-mediated GCRs relative to that of the individual single mutants (Table 4; p<0.0001). In contrast, the *rad5Δ pol30-119* double mutant had an increased rate of accumulating duplication-mediated GCRs that was indistinguishable from that of the respective single mutants (p = 0.04 for *rad5Δ* and p = 0.08 for *pol30-119*), suggesting epistasis of *RAD5* to post-translational modifications of PCNA at Lys164. Remarkably, the *rad5Δ siz1Δ* double mutant and the *rad5Δ* single mutant had indistinguishable rates of accumulating duplication-mediated GCRs, suggesting that sumoylation of PCNA at Lys164 was of limited importance for the suppression of duplication-mediated GCRs (Table 4).

### *SRS2* is epistatic to *RAD5*, and deletion of *RAD5* partially suppresses an *SGS1* deletion

Both replication fork regression [5] and cross-fork [8]–[10] template-switching mechanisms involving Rad5 helicase action would result in branched DNA structures requiring additional processing. We therefore used the sensitivity of the duplication-mediated GCR assay to deletion of *RAD5* to screen for helicases that might act in concert with *RAD5*.

Deletion of *MGS1*, *MPH1*, *RRM3*, *HRQ1* or *IRC20* did not increase the rate of single copy sequence mediated GCRs in the *yel068c::CAN1/URA3* assay and caused small, up to 6-fold, increases in the rate of duplication-mediated GCRs in the *yel072w::CAN1/URA3* assay (Table 5). Deletion of *IRC20* had no effect on the overall duplication-mediated GCR rate or the rate of any specific class of GCR (Figure 1b). The *mgs1Δ rad5Δ* and *mph1Δ rad5Δ* double mutants did not have increased rates of single copy sequence-mediated GCRs but had significantly increased rates of duplication-mediated GCRs relative to the single mutants (p = 0.0001) that were greater than additive (*mgs1Δ rad5Δ*) or at least as high as additive (*mph1Δ rad5Δ*), suggesting that Mgs1 and Mph1 may have roles in suppressing duplication mediated GCRs that are independent of Rad5. The *rrm3Δ rad5Δ*, *hrq1Δ rad5Δ* and *irc20Δ rad5Δ* double mutants did not have increased rates of single copy sequence-mediated GCRs and had increased rates of duplication-mediated GCRs that were the same as that of the *rad5Δ* single mutant and which could not be distinguished from additivity; in our view this latter double mutant analysis provided no strong evidence for epistasis because of the very small affect of *rrm3Δ*, *hrq1Δ* and *irc20Δ* single mutations on duplication-mediated GCR rates.

**Table 5:** Effects of combining defects in *RAD5* with other helicase-encoding genes on duplication-mediated GCRs.

*Genotype*	*yel068c:: CAN1/URA3*		*yel072w:: CAN1/URA3*		*Ratio*
	*RDKY Number*	Can^R^5FOA^R^ Rate*	*RDKY Number*	Can^R^5FOA^R^ Rate*	
Wild-type**	6677	2.27 [1.3−4.8]×10^−9^ (1)	6678	1.97 [1.6−4.3]×10^−8^ (8.7)	8.7
*rad5Δ*	6898	5.00 [1.4−10]×10^−9^ (2.2)	6940	3.78 [2.4−5.8]×10^−7^ (167)	76
*mgs1Δ*	6890	9.60 [3.3−37]×10^−10^ (0.4)	6932	2.45 [1.9−5.9]×10^−8^ (11)	25
*mgs1Δ rad5Δ*	6891	3.22 [1.2−7.6]×10^−9^ (1.4)	6933	1.08 [0.7−1.7]×10^−6^ (476)	337
*mph1Δ* **	6794	2.00 [0.0−17]×10^−9^ (0.9)	6795	1.05 [9.3−13]×10^−7^ (48)	54
*mph1Δ rad5Δ*	6893	2.59 [0.0−13]×10^−10^ (0.1)	6935	9.13 [6.4−14]×10^−7^ (402)	3528
*rrm3Δ*	6912	9.46 [0.0−12]×10^−10^ (0.4)	6954	3.87 [2.6−6.8]×10^−8^ (18)	43
*rrm3Δ rad5Δ*	6913	1.13 [0.7−1.4]×10^−8^ (5.0)	6955	6.55 [4.7−9.9]×10^−7^ (289)	58
*pif1Δ*	6894	3.73 [2.0−5.8]×10^−7^ (164)	6936	3.61 [2.7−5.9]×10^−7^ (159)	1.0
*pif1Δ rad5Δ*	6895	1.88 [1.5−2.8]×10^−7^ (83)	6937	4.87 [3.1−7.1]×10^−7^ (215)	2.6
*sgs1Δ* **	6687	1.69 [0.3−3.0]×10^−8^ (7.5)	6690	1.93 [1.6−2.5]×10^−6^ (850)	114
*sgs1Δ rad5Δ*	6914	1.13 [0.6−7.1]×10^−9^ (0.5)	6956	7.36 [5.4−10]×10^−7^ (324)	345
*srs2Δ* **	6741	7.18 [0.0−32]×10^−10^ (0.3)	6758	1.28 [0.6−1.6]×10^−7^ (56)	178
*srs2Δ rad5Δ*	6916	4.58 [0.0−28]×10^−10^ (0.2)	6958	1.36 [0.9−1.6]×10^−7^ (60)	297
*hcs1Δ*	6883	6.13 [0.0−31]×10^−10^ (0.3)	6925	1.22 [0.7−1.7]×10^−7^ (54)	199
*hcs1Δ rad5Δ*	6884	4.66 [0.0−2.3]×10^−10^ (0.2)	6926	1.75 [1.3−4.2]×10^−7^ (77)	374
*hrq1Δ*	6885	2.20 [0.0−5.6]×10^−10^ (0.1)	6927	6.32 [3.5−11]×10^−8^ (28)	287
*hrq1Δ rad5Δ*	6886	<8.7 [7.6−16]×10^−10^ (0.4)	6928	4.29 [3.2−4.9]×10^−7^ (189)	>490
*irc20Δ*	6887	2.64 [0.9−14]×10^−10^ (0.1)	6929	2.30 [2.1−6.2]×10^−8^ (10)	87
*irc20Δ rad5Δ*	6888	5.34 [3.8−8.6]×10^−10^ (0.2)	6930	5.99 [4.7−7.1]×10^−7^ (264)	1122

*The number in parentheses is the fold increase relative to RDKY6677. Numbers in brackets represent the 95% confidence intervals.

**Rate from [27].

A *pif1Δ* mutation caused a similar increase in the rate of GCRs in both the *yel068c::CAN1/URA3* and *yel072w::CAN1/URA3* GCR assays (Table 5); the lack of a duplication-specific increase in the GCR rate is consistent with the fact that Pif1 functions to suppress the healing of broken chromosomes by *de novo* telomere addition [48] and the fact that none of the isolates from the *yel072w::CAN1/URA3* GCR assay were homology-mediated translocations (Figure 1b). The *rad5Δ pif1Δ* double mutant had a modest decrease in the rate of single-copy sequence mediated GCRs compared to the *pif1Δ* mutant consistent with published results [49], whereas the *rad5Δ pif1Δ* double mutant had that same rate of duplication-mediated GCRs as both the *pif1Δ* and *rad5Δ* single mutants (p = 0.2). This latter result could be explained by *pif1Δ* and *rad5Δ* affecting the same pathway or by GCR-producing pathways activated by *pif1Δ* and *rad5Δ* mutations competing for the same source of broken chromosomes with one pathway being dominant.

An *srs2Δ* mutation had no affect on the rate of single copy sequence-mediated GCRs and caused an increased in the rate of duplication-mediated GCRs that was less than that caused by a *rad5Δ* mutation. The *srs2Δ rad5Δ* double mutant did not have an increased rate of single copy sequence-mediated GCRs (Table 5) but had an increased rate of duplication mediated GCRs that was the same as that of the *srs2Δ* single mutant (p = 0.6) and less than that of the *rad5Δ* single mutant (Table 5; p<0.0001). These results are consistent with previously observed epistasis between *srs2Δ* and *rad5Δ* for spontaneous recombination, triplet-repeat expansion and UV sensitivity [23], [24], [50], [51], and the partial epistasis for *srs2Δ* and *rad6Δ* observed above (Table 4). Thus it seems likely that *RAD5* might function in an *SRS2*-dependent pathway. Remarkably, mutations in *HCS1*, which encodes a DNA polymerase alpha-associated helicase [52], behaved exactly the same as *srs2Δ* mutations suggesting that an *hcs1Δ* mutation might also be epistatic with or slightly suppress a *rad5Δ* mutation in the duplication-mediated GCR assay.

The *rad5Δ sgs1Δ* double mutant had a lower rate of accumulating duplication-mediated GCRs than an *sgs1Δ* single mutant (p<0.0001), and the double mutant rate was similar to but somewhat higher than that of a *rad5Δ* single mutant (Table 5; p = 0.002). This partial epistasis of *sgs1Δ* to *rad5Δ* is consistent with a role for *SGS1* downstream of *RAD5*. The partial nature of the epistasis, however, suggests that *SGS1* also has *RAD5*-independent roles as well. Despite the indication that *RAD5* might function upstream of *SGS1*, deletion of *RAD5* did not suppress the synthetic lethality and growth defects observed between an *sgs1Δ* mutation and *srs2Δ*, *rrm3Δ*, *mus81Δ*, *slx1Δ*, *slx5Δ* or *slx8Δ* mutations nor did deletion of both *RAD5* and *RAD52* suppress the lethality between an *sgs1Δ* mutation and *slx4Δ* or *slx8Δ* mutations (not shown).

## Discussion

**Figure 2:**
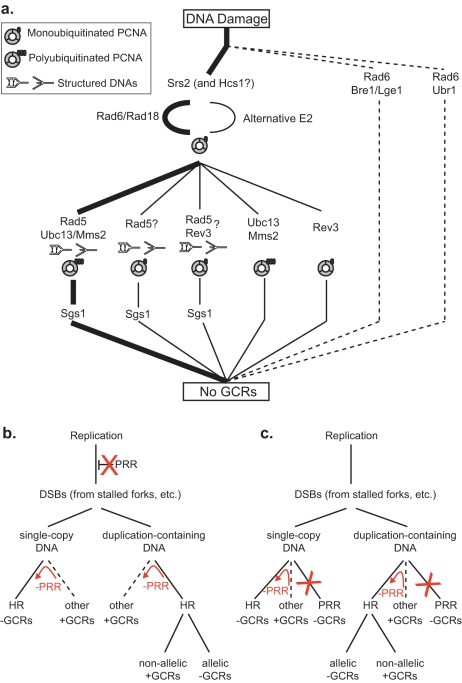
Models for the suppression of duplication-mediated GCRs by PRR. **A.** The most important *RAD6*-dependent pathway that suppresses duplication-mediated GCRs (thick lines) corresponds to the “error-free” PRR branch, which is downstream of Srs2. Other Rad6- and Rad18-dependent branches are less important (thin lines). The presence of specific PCNA and DNA states are inferred based on the genes involved in the pathway. Sgs1 appears to act downstream of the Rad5-dependent branches. The existence of Rad5 branches that are independent of Ubc13 and Rev3 that could be dependent upon Rad6 and Rad18 or independent of Rad6 is inferred by the observation of synergistic interactions between mutations in *RAD5* and mutations in *RAD6*, *UBC13* and *REV3*. Our data do not directly address the previously identified Rad5- and Rev3-dependent branch [46]. **B.** PRR could potentially suppress duplication-mediated GCRs by preventing replication damage from being converted into DSBs and other HR substrates. Suppression of single-copy GCRs also requires that PRR promotes other GCR forming pathways (such as NHEJ and *de novo* telomere addition) or requires PRR-dependent suppression of HR. **C.** PRR could potentially suppress duplication-mediated GCRs by functioning as an alternative to HR. Suppression of single-copy GCRs also requires that PRR promotes other GCR forming pathways (such as NHEJ and *de novo* telomere addition) or requires PRR-dependent suppression of HR. The red arrows and Xs in **B** and **C** indicate the consequences of PRR defects.

In the present study, we have demonstrated that suppression of duplication-mediated GCRs by *RAD6* is epistatic to the replication stress checkpoint and that the *RAD18*-, *RAD5*-, *UBC13*-, and *MMS2*-dependent error-free PRR pathway is the *RAD6*-dependent pathway that is primarily responsible for suppressing duplication-mediated GCRs. The translesion polymerase-dependent pathways for PRR and the *BRE1*- and *UBR1*-dependent *RAD6* pathways played small roles in suppressing duplication-mediated GCRs. In addition, genes that are not typically considered as encoding components of the PRR pathways, but which have been implicated in PRR by a few genetic studies, including *RAD9* [53] and *TAF14* (*ANC1*) [41], as well as the Shu complex genes, *PSY3* and *CSM2*, implicated as acting downstream of *RAD5* [54], did not appear to play significant roles in suppressing duplication-mediated GCRs (Tables 1 & 2, and not shown). The suppression of duplication-mediated GCRs exhibited remarkably complex genetic interactions between downstream PRR components (Figure 2a), involved the helicase and not the RING-finger functions of Rad5, and required Sgs1 for processing of repair intermediates. Our analysis using the sensitive duplication-mediated GCR assay revealed a number of surprising results that appear paradoxical in the context of commonly accepted models for PRR [4], but fit with a growing body of evidence that indicate that the *in vivo* pathways are more complicated than can be accounted for by present models [26], [45]–[47], [55]–[57].

The first surprising result is the lack of epistasis between *RAD5* and *UBC13* in the duplication-mediated GCR assay, as the Ubc13-Mms2 synthesis of Lys63-linked polyubiquitin chains on PCNA is dependent upon the E3 function of Rad5 [21], [57]. This lack of epistasis is consistent with a function of Rad5 that is independent of PCNA polyubiquitination, consistent with our observation of weak defects caused by mutations affecting the Rad5 RING finger function but large defects caused by mutations affecting the Rad5 helicase function, and consistent with a role of Rad5 in some translesion polymerase-dependent events [45], [46] and the ability of Rad5 to recognize and bind PCNA with a similar affinity regardless of its ubiquitination status [57]. This lack of epistasis also argues for a role of Ubc13 independent of Rad5 that would not have been predicted by the lesser sensitivity of *UBC13* and *MMS2* mutants to DNA damaging agents than seen with *rad5* mutants [45], [47], and may reconcile the weak effect of the Rad5 RING finger mutation in the duplication-mediated GCR assay with the stronger effect of the *ubc13Δ* mutation. Together with the observation that a *rev3Δ* mutation shows synergistic interactions with both *rad5Δ* and *ubc13Δ* mutations in the duplication-mediated GCR assay, this supports the idea that there are individual pathways that repair spontaneous damage that are solely dependent upon *REV3*, *RAD5* or *UBC13* in addition to pathways that are dependent upon combinations of these genes (Figure 2a); in the context of this model, the rates of duplication-mediated GCRs seen in different mutants suggest that *UBC13* and *RAD5* function in the same pathway more frequently than other combinations in accord with more simple models of “error-free” and “error-prone” branches [4].

The second surprising result from our studies is the fact that the increased duplication-mediated GCR rate caused by the *rad5Δ* mutation was not affected by a deletion of *SIZ1* but the *rad5Δ* mutation was epistatic to both a deletion of *SRS2* and the *pol30-119* mutation in the duplication-mediated GCR assay. The *SRS2* gene was originally identified through the isolation of a mutation that suppressed the trimethoprim- and UV-sensitive phenotypes of *rad6Δ* and *rad18Δ* mutants [36] where HR was required for suppression [58]. Epistasis of a *rad5Δ* mutation with a *srs2Δ* mutation is consistent with previous observations of a requirement for *SRS2* for *RAD5*-dependent error-free PRR [51], and could be due to direct recruitment of Rad5 to the site of DNA damage by Srs2 or indirect recruitment via a role of Srs2 in suppressing HR [59], [60]. Our results are not consistent, however, with an absolute requirement of Siz1-mediated PCNA sumoylation and subsequent Srs2 recruitment for Srs2 function to suppress duplication-mediated GCRs. For example, a *srs2Δ* mutation caused a greater GCR rate in the duplication-mediated GCR assay than a *siz1Δ* mutation and was strongly epistatic to PRR gene deletions, which is consistent with previously published results that an *srs2Δ* mutation causes a greater suppression of the DNA damaging agent sensitivity caused by a *rad6Δ* mutation than the level of suppression caused by a *siz1Δ* mutation [34]. The observation of Cdk1- and PCNA-independent roles of Srs2 in the completion of synthesis-dependent strand annealing [56] is also consistent with a Siz1-independent role of Srs2. However, this contrasts with suggestions of *SIZ1*-dependence of PRR based on genetic interactions between *siz1Δ* and *rad18Δ* mutations [8], [34].

The third surprising result from our studies is the synergistic interaction between the deletion of *RAD6* and the deletion of *RAD5* in the duplication-mediated GCR assay, as Rad5 is typically considered to function downstream of Rad6-Rad18-mediated monoubiquitination of PCNA at Lys163 [4], [17]. This result is even more surprising given the equivalent duplication-mediated GCR rates observed in *rad6Δ*, *rad18Δ*, *pol30-119*, and *rad6Δ pol30-119* mutants and the apparent epistasis of *rad5Δ* and *pol30-119* mutations in the duplication-mediated GCR assay. The epistasis of *pol30-119*, but not *rad6Δ*, to *rad5Δ*, and the lack of effect of combining *siz1Δ* and *rad5Δ* mutations are inconsistent with models suggesting Rad6-dependent monoubiquition of PCNA at Lys164 is absolutely required for Rad5 function. However, these results are consistent with the possibility that ubiquitin ligases other than Rad6 can modify Lys164 of PCNA *in vivo*, which has been observed to occur at low levels in *rad6Δ* mutants [26].

Extensive pathway analysis has led to the hypothesis that replication errors are a major form of spontaneous DNA damage giving rise to duplication-mediated GCRs [27]. Thus, the apparent epistasis of *RAD6* to components of the replication stress checkpoint suggests that maintaining appropriate DNA structures at the replication checkpoint [61], [62] is important for the PRR pathway to suppress duplication-mediated GCRs, and might be required for PRR to repair replication damage via template-switching pathways [6]–[10], which likely operates in competition with other pathways that might excise such DNA damage [5], [45]. Generation of potential template-switched products by the Rad5 helicase activity would produce molecules requiring further processing. We found that a *rad5Δ* mutation partially suppressed the defects of the *sgs1Δ* mutation, potentially suggesting that *RAD5*-dependent DNA structures that lead to GCRs accumulate in *sgs1Δ* mutants. This idea is consistent with the observation of HR-dependent DNA intermediates in *sgs1Δ* strains that accumulate in a *RAD5*-dependent manner [8] and the observed patterns of sensitivity to DNA damaging agents caused by different combinations of *sgs1Δ*, *mms2Δ*, and *pol30-119* mutations [8], [54]. This observed partial epistasis is also consistent with the ability of *SGS1* and its human homolog *BLM* to unwind Holliday junctions and other branched DNA structures [63]–[66] and resolve double-Holliday junctions [67]. Interestingly, we also found that *srs2Δ* and *hcs1Δ* mutations were epistatic to a *rad5Δ* mutation suggesting that the Srs2 and Hcs1 helicases may also act in processing stalled replication forks.

Our data suggest how PRR defects cause increased rates of duplication-mediated GCRs, but not single-copy sequence mediated-GCRs, yet suppress the increased rates of single-copy sequence-mediated GCRs caused by mutations in genes such as *ASF1* [26], *PIF1* ([25], Table 5) and *RAD53* (Table 1) (Figures 2b,c). These phenotypes are not simply a matter of PRR mutants having a hyperrecombination phenotype [23], [68], as other hyperrecombination mutants, such as *rad27Δ* [69], [70] and *mre11Δ*, *rad50Δ* and *xrs2Δ* mutants [71]–[74] have increased rates of both single-copy sequence- and duplication-mediated GCRs [27], [75] and likely have an increased basal level of spontaneous DNA damage. Rather, PRR must function either by preventing damage from becoming HR substrates (Figure 2b) or as an alternative pathway to HR in the processing of damage (Figure 2c). PRR defects would thus increase the potential for HR, increasing the rate of duplication-mediated GCRs resulting from non-allelic HR while having little affect or even suppressing the rate of single-copy sequence-mediated GCRs as increased allelic HR acts on single-copy sequences to suppress GCRs [25], [26]. These models are consistent with the synergisitic effects of deleting *RAD5* or *RAD18* in conjunction with deleting *RAD52* on the rate of single copy sequence-mediated GCRs as well as the decreased rates of duplication-mediated GCRs caused by deleting *RAD52* in PRR mutants. Moreover, an additional role of PRR is suggested by the fact that PRR defective mutations also suppress the high rate of single copy sequence-mediated GCRs caused by different mutations. This additional role could be indirectly or directly suppressing HR, such as by controlling the nature of damaged DNA or by the Srs2-mediated suppression of Rad51 filaments [59], [60]. Alternatively, this additional function of PRR could promote the processing of DNA damage by non-HR mediated mechanisms like non-homologous end-joining (NHEJ) or chromosome healing by *de novo* telomere addition [43], [44]. We note that the generality of PRR defective mutations in suppressing the increased rates of single copy sequence-mediated GCRs caused by different mutations has not yet been broadly established; in addition, *RAD5* has been reported to suppress NHEJ [76]. The role of PRR in specifically suppressing duplication-mediated GCRs suggests that PRR plays critical roles in suppressing non-allelic HR in genomes containing high levels of duplicated sequences. In humans, suppression of non-allelic HR is likely important for preventing genome rearrangements from occurring due to the large numbers of duplicated sequences in the human genome [77], [78] and to suppress copy number variations that contribute to human genetic variation and genetic disease [79], [80].

## Materials and Methods

### Construction and propagation of strains

Synthetic drop-out media for propagation of strains and measuring GCR rates were as described [75]. GCR assays were performed using derivatives of RDKY6678 (*yel072w::CAN1/URA3*) or RDKY6677 (*yel068c::CAN1/URA3*) that in addition have the genotype *MATa leu2Δ1 his3Δ200 trp1Δ63 lys2ΔBgl hom3-10 ade2Δ1 ade8 ura3-52 can1::hisG iYEL072::hph* as previously described as was the analysis of the structure of the resulting GCRs [27]. Mutant derivatives of these strains (Table S2) were constructed using standard PCR-based gene disruption methods as described [75].

### Statistical methods

The lower and upper bounds of 95% confidence intervals of the median were calculated as described (http://www.math.unb.ca/~knight/utility/MedInt95.htm). We calculated probabilities for the null model of the observed distributions being generated by the same underlying rate using the two-tailed Mann-Whitney U-test (http://faculty.vassar.edu/~lowry/utest.html). Statistically significant differences in rates were taken to be cases where the probability of the null model was 0.01 or less.

## Supporting Information

Table S1 Recovery of hygromycin resistant GCRs.
(0.08 MB PDF)

Table S2 Yeast strains.
(0.08 MB PDF)
