# Supplementary material for: Post-Replication Repair Suppresses Duplication-Mediated Genome Instability
Source: PLoS Genet. 2010 May 6;6(5):e1000933. doi: 10.1371/journal.pgen.1000933 (PMC2865514; doi:10.1371/journal.pgen.1000933)
Supplement: Table S1 — Recovery of hygromycin resistant GCRs. (0.08 MB PDF) [file pgen.1000933.s001.pdf]

Table S1. Frequency for recovery of hygromycin resistant GCRs.

Genotype	***yel068c::CAN1/URA3***		***yel072w::CAN1/URA3***	
	Percentage hph+	***Number hph+***	***Percentage hph+***	***Number hph+***
Wild-type	7.4%	2 of 27	0%	0 of 62
bre1	0%	0 of 9	0%	0 of 14
hcs1	0%	0 of 8	0%	0 of 14
hcs1 rad5	0%	0 of 9	0%	0 of 7
hrq1	0%	0 of 4	0%	0 of 14
hrq1 rad5	0%	0 of 3	0%	0 of 14
irc20	20%	1 of 5	0%	0 of 14
irc20 rad5	0%	0 of 8	0%	0 of 14
lge1	0%	0 of 2	0%	0 of 14
mec1 sml1	0%	0 of 14	0%	0 of 14
mgs1	0%	0 of 2	0%	0 of 14
mgs1 rad5	0%	0 of 14	0%	0 of 14
mph1	0%	0 of 8	0%	0 of 14
mph1 rad5	0%	0 of 7	0%	0 of 14
mms2	8.3%	1 of 12	0%	0 of 14
mrc1	14.3%	1 of 7	0%	0 of 14
mrc1-aq	0%	0 of 7	0%	0 of 14
pif1	0%	0 of 14	0%	0 of 14
pif1 rad5	0%	0 of 14	0%	0 of 14
pol30-119	7.7%	1 of 13	0%	0 of 14
pol30-119 rad5	0%	0 of 13	0%	0 of 14
pol30-119 rad6	0%	0 of 3	0%	0 of 14
rad5	15.4%	2 of 13	0%	0 of 14
rad5 rad52	0%	0 of 14	0%	0 of 14
rad18	42.9%	3 of 7	0%	0 of 11
rad18 rad52	0%	0 of 14	0%	0 of 14
rad52	0%	0 of 12	0%	0 of 55
rad53 sml1	21.4%	3 of 14	0%	0 of 14
rad6	0%	0 of 1	0%	0 of 52
rad6 mrc1	18.2%	2 of 11	0%	0 of 14

rad6 mrc1-aq	0%	0 of 2	0%	0 of 14
rad6 tof1	16.7%	1 of 6	0%	0 of 14
rad6 mec1 sml1	0%	0 of 14	0%	0 of 14
rad6 rad53 sml1	8.3%	1 of 12	0%	0 of 14
rad6 rad9	25.0%	2 of 8	0%	0 of 14
rad6 rad5	0%	0 of 11	0%	0 of 14
rad9	0%	0 of 11	0%	0 of 14
rev3	0%	0 of 9	0%	0 of 14
rev3 rad5	0%	0 of 6	0%	0 of 14
rev3 rad30	0%	0 of 9	0%	0 of 14
rev3 ubc13	14.3%	2 of 14	7.1%	1 of 14
rad30	18.2%	2 of 11	0%	0 of 14
rrm3	25.0%	2 of 8	7.1%	1 of 14
rrm3 rad5	0%	0 of 14	0%	0 of 14
sgs1	21.4%	3 of 14	0%	0 of 54
sgs1 rad5	0%	0 of 13	0%	0 of 13
siz1	0%	0 of 2	0%	0 of 14
siz1 rad5	0%	0 of 11	0%	0 of 14
srs2	0%	0 of 3	0%	0 of 14
srs2 rad5	0%	0 of 9	0%	0 of 14
srs2 rad6	0%	0 of 1	0%	0 of 14
tsa1	0%	0 of 14	14.3%	2 of 14
tsa1 rad30	23.1%	3 of 13	0%	0 of 14
tsa1 rev3	21.4%	3 of 14	0%	0 of 14
taf14	0%	0 of 2	0%	0 of 12
tof1	33.3%	1 of 3	0%	0 of 14
ubc13	11.1%	1 of 9	0%	0 of 14
ubc13 rad5	0%	0 of 2	0%	0 of 14
ubr1	0%	0 of 2	0%	0 of 18
**Total**	**7.1%**	**36 of 509**	**0.44%**	**4 of 940**
