# Supplementary material for: Post-Replication Repair Suppresses Duplication-Mediated Genome Instability
Source: PLoS Genet. 2010 May 6;6(5):e1000933. doi: 10.1371/journal.pgen.1000933 (PMC2865514; doi:10.1371/journal.pgen.1000933)
Supplement: Table S2 — Yeast strains. (0.08 MB PDF) [file pgen.1000933.s002.pdf]

Table S2. Yeast strains.

Name	Genotype
RDKY6677	*MATa leu2Δ1 trp1Δ63 his3Δ200 lys2ΔBgl hom3-10 ade2Δ1 ade8 ura3-52 iYEL072W::hph can1::hisG yel068c::CAN1/URA3*
RDKY6678	*MATa leu2Δ1 trp1Δ63 his3Δ200 lys2ΔBgl hom3-10 ade2Δ1 ade8 ura3-52 iYEL072W::hph can1::hisG yel072w::CAN1/URA3*
RDKY6882	RDKY6677 *bre1::HIS3*
RDKY6883	RDKY6677 *hcs1::HIS3*
RDKY6884	RDKY6677 *hcs1::HIS3 rad5::G418*
RDKY6885	RDKY6677 *hrq1::HIS3*
RDKY6886	RDKY6677 *hrq1::HIS3 rad5::G418*
RDKY6887	RDKY6677 *irc20::HIS3*
RDKY6888	RDKY6677 *irc20::HIS3 rad5::G418*
RDKY6889	RDKY6677 *lge1::HIS3*
RDKY6760	RDKY6677 *mec1::HIS3 sml1::G418*
RDKY6890	RDKY6677 *mgs1::HIS3*
RDKY6891	RDKY6677 *mgs1::HIS3 rad5::G418*
RDKY6892	RDKY6677 *mms2::TRP1*
RDKY6794	RDKY6677 *mph1::HIS3*
RDKY6893	RDKY6677 *mph1::HIS3 rad5::G418*
RDKY6730	RDKY6677 *mrc1::TRP1*
RDKY6766	RDKY6677 *mrc1-aq.TRP1*
RDKY6894	RDKY6677 *pif1::HIS3*
RDKY6895	RDKY6677 *pif1::HIS3 rad5::G418*
RDKY6896	RDKY6677 *pol30-119.LEU2*
RDKY6897	RDKY6677 *pol30-119.LEU2 rad5::G418*
RDKY7033	RDKY6677 *pol30-119.LEU2 rad6::NAT*
RDKY6898	RDKY6677 *rad5::HIS3*
RDKY6899	RDKY6677 *rad5::HIS3 rad52::HIS3*
RDKY6733	RDKY6677 *rad6::HIS3*
RDKY6900	RDKY6677 *rad6::NAT mec1::HIS3 sml1::G418*
RDKY6901	RDKY6677 *rad6::HIS3 mrc1::TRP1*
RDKY6966	RDKY6677 *rad6::HIS3 mrc1-aq.TRP1*
RDKY6902	RDKY6677 *rad6::HIS3 rad5::G418*
RDKY6903	RDKY6677 *rad6::NAT rad9::HIS3*
RDKY6904	RDKY6677 *rad6::NAT rad53::HIS3 sml1::G418*
RDKY6968	RDKY6677 *rad6::NAT tof1::HIS3*
RDKY6765	RDKY6677 *rad9::HIS3*
RDKY6905	RDKY6677 *rad18::G418*

**Name**	**Genotype**
RDKY6906	RDKY6677 *rad18::G418 rad52::HIS3*
RDKY6907	RDKY6677 *rad30::G418*
RDKY6691	RDKY6677 *rad52::HIS3*
RDKY6762	RDKY6677 *rad53::HIS3 sml1::G418*
RDKY6908	RDKY6677 *rev3::HIS3*
RDKY6909	RDKY6677 *rev3:HIS3 rad5::G418*
RDKY6910	RDKY6677 *rev3::HIS3 rad30::G418*
RDKY6911	RDKY6677 *rev3::HIS3 ubc13::TRP1*
RDKY6912	RDKY6677 *rrm3::TRP1*
RDKY6913	RDKY6677 *rrm3::TRP1 rad5::G418*
RDKY6687	RDKY6677 *sgs1::HIS3*
RDKY6914	RDKY6677 *sgs1::HIS3 rad5::G418*
RDKY6915	RDKY6677 *siz1::TRP1*
RDKY7035	RDKY6677 *siz1::TRP1 rad5::G418*
RDKY6741	RDKY6677 *srs2::HIS3*
RDKY6916	RDKY6677 *srs2::HIS3 rad5::G418*
RDKY7034	RDKY6677 *srs2::HIS3 rad6::NAT*
RDKY6917	RDKY6677 *taf14::HIS3*
RDKY6767	RDKY6677 *tof1::HIS3*
RDKY6918	RDKY6677 *tsa1::G418*
RDKY6919	RDKY6677 *tsa1::G418 rad30::HIS3*
RDKY6920	RDKY6677 *tsa1::G418 rev3:HIS3*
RDKY6921	RDKY6677 *ubc13::TRP1*
RDKY6922	RDKY6677 *ubc13::TRP1 rad5::G418*
RDKY6923	RDKY6677 *ubr1::NAT*
RDKY6924	RDKY6678 *bre1::HIS3*
RDKY6925	RDKY6678 *hcs1::HIS3*
RDKY6926	RDKY6678 *hcs1::HIS3 rad5::G418*
RDKY6927	RDKY6678 *hrq1::HIS3*
RDKY6928	RDKY6678 *hrq1::HIS3 rad5::G418*
RDKY6929	RDKY6678 *irc20::HIS3*
RDKY6930	RDKY6678 *irc20::HIS3 rad5::G418*
RDKY6931	RDKY6678 *lge1:HIS3*
RDKY6769	RDKY6678 *mec1::HIS3 sml1::G418*
RDKY6932	RDKY6678 *mgs1::HIS3*
RDKY6933	RDKY6678 *mgs1::HIS3 rad5::G418*
RDKY6934	RDKY6678 *mms2::TRP1*
RDKY6795	RDKY6678 *mph1::HIS3*

Name	Genotype
RDKY6935	RDKY6678 *mph1::HIS3 rad5::G418*
RDKY6747	RDKY6678 *mrc1::TRP1*
RDKY6775	RDKY6678 *mrc1-aq.TRP1*
RDKY6936	RDKY6678 *pif1::HIS3*
RDKY6937	RDKY6678 *pif1::HIS3 rad5::G418*
RDKY6938	RDKY6678 *pol30-119.LEU2*
RDKY6939	RDKY6678 *pol30-119.LEU2 rad5::G418*
RDKY7036	RDKY6678 *pol30-119.LEU2 rad6::NAT*
RDKY6940	RDKY6678 *rad5::HIS3*
RDKY6941	RDKY6678 *rad5::G418 rad52::HIS3*
RDKY6750	RDKY6678 *rad6::HIS3*
RDKY6942	RDKY6678 *rad6::NAT mec1::HIS3 sml1::G418*
RDKY6943	RDKY6678 *rad6::HIS3 mrc1::TRP1*
RDKY6967	RDKY6678 *rad6::HIS3 mrc1-aq.TRP1*
RDKY6944	RDKY6678 *rad6:::HIS3 rad5::G418*
RDKY6945	RDKY6678 *rad6::NAT rad9::HIS3*
RDKY6946	RDKY6678 *rad6::NAT rad53::HIS3 sml1::G418*
RDKY6969	RDKY6678 *rad6::NAT tof1::HIS3*
RDKY6774	RDKY6678 *rad9::HIS3*
RDKY6947	RDKY6678 *rad18::G418*
RDKY6948	RDKY6678 *rad18::G418 rad52::HIS3*
RDKY6949	RDKY6678 *rad30::G418*
RDKY6708	RDKY6678 *rad52::HIS3*
RDKY6771	RDKY6678 *rad53::HIS3 sml1::G418*
RDKY6950	RDKY6678 *rev3::HIS3*
RDKY6951	RDKY6678 *rev3::HIS3 rad5::G418*
RDKY6952	RDKY6678 *rev3::HIS3 rad30::G418*
RDKY6953	RDKY6678 *rev3::HIS3 ubc13::TRP1*
RDKY6954	RDKY6678 *rrm3::TRP1*
RDKY6955	RDKY6678 *rrm3::TRP1 rad5::G418*
RDKY6690	RDKY6678 *sgs1::HIS3*
RDKY6956	RDKY6678 *sgs1::HIS3 rad5::G418*
RDKY6957	RDKY6678 *siz1::TRP1*
RDKY7038	RDKY6678 *siz1::TRP1 rad5::G418*
RDKY6758	RDKY6678 *srs2::HIS3*
RDKY6958	RDKY6678 *srs2::HIS3 rad5::G418*
RDKY7037	RDKY6678 *srs2::HIS3 rad6::NAT*
RDKY6959	RDKY6678 *taf14::HIS3*

Name	Genotype
RDKY6776	RDKY6678 *tof1::HIS3*
RDKY6960	RDKY6678 *tsa1::G418*
RDKY6961	RDKY6678 *tsa1::G418 rad30::HIS3*
RDKY6962	RDKY6678 *tsa1::G418 rev3::HIS3*
RDKY6963	RDKY6678 *ubc13::TRP1*
RDKY6964	RDKY6678 *ubc13::TRP1 rad5::G418*
RDKY6965	RDKY6678 *ubr1::NAT*
